# Open Access to Large Scale Datasets Is Needed to Translate Knowledge of Cancer Heterogeneity into Better Patient Outcomes

**DOI:** 10.1371/journal.pmed.1001794

**Published:** 2015-02-24

**Authors:** Andrew H. Beck

**Affiliations:** Cancer Research Institute, Beth Israel Deaconess Cancer Center, Department of Pathology, Beth Israel Deaconess Medical Center, Harvard Medical School, Boston, Massachusetts, United States of America

## Abstract

In this guest editorial, Andrew Beck discusses the importance of open access to big data for translating knowledge of cancer heterogeneity into better outcomes for cancer patients.

Cancer is a heterogeneous disease, which is comprised of a collection of diseases traditionally categorized by tissue type of origin. A distinct set of etiologic causes, treatments, and prognoses are associated with different cancers, and even within a given tissue type, cancer shows significant variability in molecular and clinical features across patients. This interpatient heterogeneity is a major rationale for large-scale research efforts (such as The Cancer Genome Atlas [TCGA] and the International Cancer Genome Consortium [ICGC]) to comprehensively profile the molecular landscape of patient cancer samples across all major cancers [1,2]. These efforts have been bolstered by the recent development of new genomic [3] and computational [4] technologies to enable increasingly detailed and comprehensive analyses of the molecular landscape of solid cancers. It is hoped that the comprehensive molecular characterization of large sets of cancer samples will lead to the identification of new therapeutic targets and the development of improved personalized therapies for cancer patients.

A major challenge in cancer therapy is the development of resistance to molecularly targeted therapies. Although targeted therapies may show initial benefit in the subset of patients carrying a targeted molecular alteration, most patients will nevertheless go on to develop resistance for most advanced solid cancers. Identifying and overcoming drug resistance represents one of the most significant challenges facing cancer researchers today [5]. It is increasingly recognized that cancer is not only a heterogeneous disease across patients but also a heterogeneous disease within individual patients, with different regions of a tumor showing different molecular features at the DNA, RNA, and protein levels [6–9]. This intratumoral molecular heterogeneity is hypothesized to be a major cause of drug resistance and treatment failure in cancer [10]. However, the clinical significance of intratumoral molecular heterogeneity is not yet well-defined, and assessment of intratumoral molecular heterogeneity is not currently used in clinical cancer medicine for assessing disease prognosis or guiding therapy. Two recent research articles published in *PLOS Medicine* show the potential clinical utility of measuring intratumoral genetic heterogeneity in clinical cancer samples.

In one, James Brenton, Florian Markowetz, and colleagues applied the Minimum Event Distance for Intra-tumour Copy-number Comparisons (MEDICC) algorithm they recently developed for phylogenetic quantification of intratumoral genetic heterogeneity from multiregion DNA copy number profiling data [11] to predict treatment resistance in high-grade serous ovarian cancer [12]. Their analysis suggests that multiregion tumor sampling, DNA copy number profiling, and quantification of intratumoral genetic heterogeneity with the MEDICC algorithm could be a useful approach for predicting patient survival in ovarian cancer, in which higher levels of heterogeneity associated with decreased survival. This study provides data to support the long-standing hypothesis regarding treatment resistance and intratumoral genetic heterogeneity [10]. Although these results are promising, the developed approach requires sampling multiple distinct regions of tumor, which would be more expensive and complex than molecular profiling from a single tissue sample. It is not yet known how much tumor sampling will be required to adequately quantify intratumoral heterogeneity in the clinic or if measuring intratumoral heterogeneity from multiple tumor samples will outperform other molecular approaches (e.g., prognostic expression signatures [13,14]) for predicting response to therapy in ovarian cancer. These are important research questions that will need to be answered prior to clinical translation.

The second study comes from James Rocco and colleagues [15]. Previously, these investigators used a publicly available data set of whole exome sequencing data in head and neck squamous cell carcinoma (HNSCC) from Stransky et al. [16] to develop a simple quantitative measure of intratumoral heterogeneity (mutant-allele tumor heterogeneity [MATH]) and showed that MATH scores were higher in poor outcome classes of HNSCC [17]. In the current study, the authors used publicly available whole exome sequencing data provided by TCGA and showed that the MATH score is associated with prognosis in HNSCC and contributes additional prognostic information beyond that provided by traditional clinical and molecular features. Since the MATH score can be computed from whole exome sequencing data obtained from a single tumor sample (which is a data type that can be obtained from formalin-fixed, paraffin-embedded tumor tissue, as is routinely collected in pathology laboratories [18]), this approach may be more easily translated into clinical use, as compared with approaches requiring multiregion sampling and more complex computational algorithms for the assessment of intratumoral heterogeneity. Nonetheless, establishing the utility of the MATH score as an effective prognostic and/or predictive biomarker in HNSCC will require additional studies of the MATH score on well-controlled clinical cohorts comprised of homogeneously treated patients with tumors at specific head and neck anatomic locations. It is important to note that the development and application of MATH for assessing prognosis in HNSCC was based entirely on the analysis of publically available clinically annotated whole exome sequencing data, which demonstrates the value in making these data open to the community.

The continuing generation of high-quality, open-access Omics data sets from large populations of cancer patients will be critically important to enable the development of computational methods to translate knowledge of cancer heterogeneity into new diagnostics and improved clinical outcomes for cancer patients. As one step towards this goal, the DREAM (Dialogue for Reverse Engineering Assessments and Methods) consortium will use open innovation crowd sourcing to identify top-performing computational methods for inferring genetic heterogeneity from next-generation sequencing data provided by a large multi-institutional community of cancer genomics projects, including the ICGC and TCGA [19]. If successful, this open innovation competition may identify a set of best-in-class methods for measuring intratumoral genetic heterogeneity in cancer.

In parallel with these advances in computational methods for inferring intratumoral heterogeneity from genomics data, genomics technologies for measuring intratumoral heterogeneity at increasingly fine levels of granularity continue to improve. For example, recent advances in single-cell sequencing of DNA have provided detailed portraits of intratumoral genetic heterogeneity and clonal evolution in cancer [20,21], and recent advances in single-cell RNA sequencing [22], in situ RNA sequencing [23,24], and highly multiplexed next-generation immunohistochemistry [25–28] enable characterization of intratumoral heterogeneity in gene expression at a single cell level with subcellular resolution. Thus, there are now many options—both molecular and computational—for measuring and analyzing intratumoral molecular heterogeneity from clinical cancer samples.

Establishing the clinical utility of these new approaches for measuring intratumoral molecular heterogeneity will require applying these methods to large sets of archival tumor samples from randomized trials of cancer therapeutics [29] and high-quality prospective observational studies [30]. To maximize the value of the data that would be produced from such an undertaking, it is critical that infrastructure be created and supported to enable sharing of the Omics and clinical data with a large community of cancer researchers and data scientists. Ensuring open access to high-quality datasets will ensure that the largest possible community of researchers is able to address the most important problems in cancer medicine today. And in generating and sharing these data widely, we will massively increase our chances of effectively translating knowledge of intratumoral heterogeneity into meaningful advances for cancer patients. 

## Abbreviations:

DREAM: Dialogue for Reverse Engineering Assessments and Methods
HNSCC: head and neck squamous cell carcinoma
ICGC: International Cancer Genome Consortium
MATH: mutant-allele tumor heterogeneity
MEDICC: Minimum Event Distance for Intra-tumour Copy-number Comparisons
TCGA: The Cancer Genome Atlas
